# Prediction of Soluble Solids and Lycopene Content of Processing Tomato Cultivars by Vis-NIR Spectroscopy

**DOI:** 10.3389/fnut.2022.845317

**Published:** 2022-06-28

**Authors:** Márton Égei, Sándor Takács, Gábor Palotás, Gabriella Palotás, Péter Szuvandzsiev, Hussein Gehad Daood, Lajos Helyes, Zoltán Pék

**Affiliations:** ^1^Institute of Horticultural Science, Hungarian University of Agriculture and Life Sciences, Gödöllő, Hungary; ^2^Univer Product PLC, Kecskemét, Hungary; ^3^Regional Knowledge Center, Hungarian University of Agriculture and Life Sciences, Gödöllő, Hungary

**Keywords:** tomato, Vis-NIR, spectroscopy, SSC, lycopene, absorbance, reflectance, preprocessing

## Abstract

Tomato-based products are significant components of vegetable consumption. The processing tomato industry is unquestionably in need of a rapid definition method for measuring soluble solids content (SSC) and lycopene content. The objective was to find the best chemometric method for the estimation of SSC and lycopene content from visible and near-infrared (Vis-NIR) absorbance and reflectance data so that they could be determined without the use of chemicals in the process. A total of 326 Vis-NIR absorbance and reflectance spectra and reference measurements were available to calibrate and validate prediction models. The obtained spectra can be manipulated using different preprocessing methods and multivariate data analysis techniques to develop prediction models for these two main quality attributes of tomato fruits. Eight different method combinations were compared in homogenized and intact fruit samples. For SSC prediction, the results showed that the best root mean squared error of cross-validation (RMSECV) originated from raw absorbance (0.58) data and with multiplicative scatter correction (MSC) (0.59) of intact fruit in Vis-NIR, and first derivatives of reflectance (*R*^2^ = 0.41) for homogenate in the short-wave infrared (SWIR) region. The best predictive ability for lycopene content of homogenate in the SWIR range (*R*^2^ = 0.47; RMSECV = 17.95 mg kg^–1^) was slightly lower than that of Vis-NIR (*R*^2^ = 0.68; 15.07 mg kg^–1^). This study reports the suitability of two Vis-NIR spectrometers, absorbance/reflectance spectra, preprocessing methods, and partial least square (PLS) regression to predict SSC and lycopene content of intact tomato fruit and its homogenate.

## Introduction

Tomato (*Lycopersicon esculentum* Mill.) and tomato-based products are significant components of vegetable consumption. The volume of processed tomatoes in 2020 exceeded 38 million tons in the world (1). From a processing point of view, the two most important quality attributes of tomato fruits are soluble solids content (SSC) and lycopene content (2, 3).

The highest cost of compaction is the energy used to evaporate water from the raw material to concentrate it to 28–38°Brix, which results in a product that can be transported more easily in this form and is eligible for further processing. Thus, when it comes to the SSC level of raw tomato, the higher SSC, the less water has to be evaporated from it, reducing the cost and energy consumption of this operation (4). This means that the processing industry pays extra price for raw tomatoes above a certain level of SSC (5).

In general, the total dry matter (DM) and SSC of the fruit increase as it ripens, in parallel with their pigmentation. The color of red-fruit varieties makes it easy to distinguish whether they are ripe or not (6–8). While SSC can be easily measured by refractometer in°Brix (9), its estimation based only on maturity depends on the cultivar (10). Therefore, it is of great importance to develop a non-destructive method for the accurate estimation of SSC of intact tomato fruits (11–14) or rapid monitoring of their homogenates in analytical laboratory or during the quality check of incoming raw material at the receiving area of processing plant (15, 16).

Lycopene, the main carotenoid component of red ripe tomato fruit, is often considered the main preference of consumers’ acceptance and a major factor for cardiovascular protection in addition to its importance in the reduction of oxidative stress active substances (17, 18).

There are examples for the estimation of lycopene content of red-ripe fruits based on their color (19, 20), but accurate values can only be obtained by expensive and time-consuming laboratory analytics (21–24). There is demand for making this procedure quicker, cheaper, and easier. Measuring spectral reflectance can be a good option for this approach (25–29).

The method is based on the near-infrared (NIR) absorption of the overtones and combination bands of water and organic molecules, mainly O–H, C–H, N–H, and C = O groups. NIR spectra are complex and more difficult to interpret as in other spectral regions like visible (Vis) spectra (30, 31).

Recently, visible-near infrared (Vis-NIR) spectroscopy has been increasingly used in studies for non-destructive determination of ingredients of fruits (32–36) and especially of tomato (22, 23, 37, 38). The rapid determination of SSC in an intact fruit or in a sample homogenized from it is a difficult task due to its high water content (39). A number of usable calibrations have already been made for the rapid determination of SSC and lycopene content in paste from processing tomatoes as the material concentrated to a°Brix value of 28–38 already contains less water (40, 41).

The development of a NIR calibration is a complex task that involves spectral collection using a NIR device, chemometrics, spectra pretreatment, calibration model development, and model validation.

The objective of this study was to evaluate the use of Vis-NIR range of spectra for measuring the SSC and lycopene content of tomato fruit and its homogenate. Choosing the best preprocessing and calibration method for the validation of these important parameters can contribute for developing a non-destructive way, which is applicable to measure these parameters quickly and accurately.

## Materials and Methods

### Plant Material

Fruits were produced in open-field experiments of processing tomato in 2019 and 2020. These experiments were carried out at the Experimental Farms of the Hungarian University of Agriculture and Life Sciences (S1), Gödöllő (47°34′N. 19°22′E; elevation 231 m) and Szarvas (S2) (46°53′N. 20°31′E; elevation 81 m) in 2019, and Experimental Farms of the Univer Agro Kft in Szentkirály (S3) (46°54′N. 19°59′E; elevation 91 m) in 2020. Production technology was the same as our previous processing tomato experiments (42–44). There were different processing tomato hybrids from three different seed companies: HeinzSeed (Pomodoro Agro Kft., Mezőberény, Hungary): H1015, H1281, H1307, H1765, H1776, H1879, H1884; BASF Nunhems (Nunhems Hungary Kft., Budapest, Hungary): N6438, NUN283, NUN287, NUN507, NUN812, NUN912, Ussar; and United Genetics (Orosco Kft., Orosháza, Hungary): UG812J, UG1410, UG5202, UG8114, UG13577, UG13579, UG14014, Prestomech. H1015 F1 and UG812J were used in both years only. Samples were formulated from 10 healthy fruits with similar visual appearance in four repetitions of each treatment combinations. The fruits were harvested by hand in red ripe stage in August of both years.

### Spectral Acquisition of the Sample’s Reflectance

Spectral and analytical measurements were performed with tomato samples right after harvesting. For the intact tomato samples, data are output as reflectance only, by ASD, because Perten is inappropriate for measuring intact fruits as its sample container does not fit intact fruit size. The reflectance and absorbance data were obtained from the laboratory of Regional Knowledge Centre of Hungarian University of Agriculture and Life Sciences (Gödöllő). For the spectral acquisition, the tomato samples were used in two forms, namely, intact tomato fruits (*n* = 132) and homogenates (*n* = 192). In the first step, the intact tomato fruits were cleaned before the collection of spectra. Spectral measurements were taken with two instruments, namely, ASD FieldSpec HandHeld 2™ (Analytical Spectral Devices. Inc., Co., United States) Portable Spectroradiometer (spectral range: 325–1,075 nm) and Perten DA7200 (Perten Instruments, Forr-Lab Kft., Budapest, Hungary) NIR analyzer (spectral range: 950–1,650 nm).

A total of 132 spectral samples were directly acquired in the range of 325–1,075 nm from S1 site in 2019 using the ASD spectroradiometer. Fruit samples were derived from irrigation and microbiological treatment combinations of H1015 and UG812J processing tomato hybrids (45).

After measurement of intact fruits, a total of 1,920 tomato fruits were washed, cut, and homogenized for the 192 samples from S2 and S3 sites in 2020. A black Teflon plate (diameter 75 mm) was filled with 26 ± 1 mm of samples, ASD spectroradiometer positioned 2 mm above samples, with Hi-Brite Contact Probe (Analytical Spectral Devices. Inc., Co., United States). Light source of the device was halogen bulb with color temperature 2,900 K, using Zenith Polymer^®^ reference panel made of sintered polytetrafluoroethylene (SphereOptics GmbH, Uhldingen, Germany) for calibration. The spectral scanning was made in five replicates. The instrument has a spectral resolution of < 3.0 nm at 700 nm and wavelength accuracy of ± 1 nm. The black plate perfectly fit into the Perten DA7200 rotation cup, the instrument was worked in the 950–1,650 nm spectral range, and the spectral resolution was 5 nm. For further spectral analysis, an average of five reflectance and absorbance recordings from each sample was used.

### Analytics

#### Soluble Solid Content

Mettler-Toledo Easy R40 refractometer (Mettler Toledo Kft., Budapest, Hungary) was used to measure the SSC of homogenized tomato samples in each replicate (10 fruits) with temperature control on 20°C (46). Its integrated Peltier temperature control quickly heats up or cools down the measurement cell, maintaining the sample reliably at the desired temperature. The tomato homogenate was filtered with gauze and dripped on the measuring cell. An average of two measurements from each repetition of samples were used for the models.

#### Lycopene

The sample was made from homogenization of 10 fruits. The sample preparation was conducted according to Daood et al. (47). Hitachi Chromaster HPLC (VWR International Kft., Debrecen, Hungary) using a Model 5110 Pump, a Model 5430 Diode Array Detector, and a Model 5210 auto-sampler. The separation and data processing were operated using the EZChrom Elite software (Agilent Technologies, Inc., Santa Clara, CA, United States). Carotenoids were detected between 190 and 700 nm. Separation of carotenoids was performed on a core C-30, 150 × 4.6 mm, 2.6 μm (Thermo Scientific, Waltham, MA, United States) column with a gradient elution of (A) tert-butyl-methyl ether in (B) 2% water in methanol (48). The gradient started with 3% A in B, changed to 35% A in B in 20 min, steady isocratic for 5 min, and finally turned to 3% A in B in 5 min. The flow rate was 0.6 ml min^–1^. For quantification, the area of each compound was recorded at the maximum absorbance wavelength. Concentration of carotenoids was calculated as 8-apo-carotenal equivalent. The internal standard was set at a known concentration to the samples. Standard material for lycopene (Sigma-Aldrich, Budapest, Hungary) was also used, as an external standard, for its identification and quantitation.

#### Spectral Data Analysis

The spectral data were analyzed using the Unscrambler 11.0 software (CAMO Analytics AS., Oslo, Norway). Preprocessing of spectral data is often of vital importance if reasonable results are to be obtained whether the analysis is used for exploratory data mining, classification, or building a good robust prediction model (49). The obtained spectra can be manipulated using different preprocessing methods and multivariate data analysis techniques to develop prediction models for these two main quality attributes of tomato fruits. Three preprocessing methods were used to improve the quality of original spectra, multiplicative scatter correction (MSC), standard normal variate (SNV), Savitzky-Golay based on first derivative (1DER) as in previous studies (32, 50, 51), and their combinations with reflectance and absorbance spectra. Partial least-square regression (PLSR) was used to develop calibration models between spectral data and SSC or lycopene content of tomatoes. Eight different method combinations were compared in fruit and homogenized samples (Table 1). The calibration set was 75%, and the validation set was 25% of the total samples. The correct number of regression factors for the PLSR model was judged by root mean square error of cross-validation (RMSECV) obtained by 10-fold cross-validation.

**TABLE 1:** Predictive capability of calibration models for SSC and lycopene content of tomato samples by ASD and Perten spectrometers.

		Fruit (*n* = 132)				Homogenate (*n* = 192)							
		ASD (Vis-NIR)				ASD (Vis-NIR)				Perten (SWIR)			
		R^2^CAL	RMSEC	R^2^VAL	RMSECV	R^2^CAL	RMSEC	R^2^VAL	RMSECV	R^2^CAL	RMSEC	R^2^VAL	RMSECV
SSC	Reflectance	0.73	0.49	0.62	0.64	0.17	0.70	0.20	0.59	0.65	0.45	0.55	0.44
	Absorbance	**0.87**	**0.35**	**0.68**	**0.58**	0.61	0.48	0.58	0.43	0.67	0.44	0.58	0.43
	REF + MSC	0.67	0.55	0.54	0.70	0.01	0.76	0.00	0.66	0.59	0.49	0.57	0.43
	REF + SNV	0.63	0.58	0.52	0.71	0.38	0.60	0.52	0.46	0.64	0.45	0.60	0.42
	REF + 1DER	0.47	0.69	0.47	0.74	0.30	0.64	0.37	0.52	**0.70**	**0.42**	**0.61**	**0.41**
	ABS + MSC	**0.88**	**0.34**	**0.72**	**0.59**	0.62	0.47	0.54	0.45	0.60	0.49	0.56	0.44
	ABS + SNV	0.85	0.37	0.66	0.60	**0.64**	**0.46**	**0.58**	**0.43**	0.66	0.45	0.59	0.42
	ABS + 1DER	0.77	0.46	0.55	0.69	0.57	0.50	0.51	0.46	**0.70**	**0.42**	**0.60**	**0.42**
LYCOPENE	Reflectance	0.36	41.01	0.42	41.06	0.07	27.38	0.10	23.47	0.46	20.99	0.43	18.70
	Absorbance	0.30	42.94	0.40	41.63	**0.86**	**10.56**	**0.68**	**15.07**	0.48	20.47	0.44	18.41
	REF + MSC	0.54	34.89	0.41	41.19	0.01	28.37	0.00	24.68	0.46	20.94	0.38	19.48
	REF + SNV	0.57	33.70	0.41	41.34	0.52	19.79	0.52	17.07	0.45	21.15	0.37	19.51
	REF + 1DER	0.50	36.37	0.28	45.68	0.26	24.47	0.20	22.10	0.44	21.21	0.42	18.77
	ABS + MSC	**0.64**	**30.62**	**0.46**	**39.64**	0.61	17.66	0.52	17.09	0.49	20.34	0.38	19.50
	ABS + SNV	**0.66**	**30.14**	**0.44**	**40.31**	0.63	17.25	0.52	17.14	0.45	21.05	0.35	19.84
	ABS + 1DER	0.52	35.50	0.34	43.64	0.46	20.98	0.41	18.99	**0.51**	**19.86**	**0.47**	**17.95**

*ASD, ASD FieldSpec HandHeld 2™ portable spectroradiometer; PERTEN, Perten DA7200 NIR analysis system; Vis-NIR, visible and near infrared; SWIR, short-wave infrared; CAL, calibration; VAL, validation; RMSEC, root mean square error of calibration; RMSECV, root mean square error of cross-validation; REF, reflectance; ABS, absorbance; MSC, multiplicative scattering correction; SNV, standard normal variate; 1DER, first derivative. Bold numbers mean the best calibration and prediction of models.*

Reflectance data were obtained from intact tomato fruits (*n* = 132) by ASD only, while from homogenized samples (*n* = 192) reflectance and absorbance were measured by ASD and Perten, respectively. One of the four replicates of each individual tomato groups was selected to represent the entire population for validation.

## Results and Discussion

### Spectral Acquisition

**FIGURE 1:**
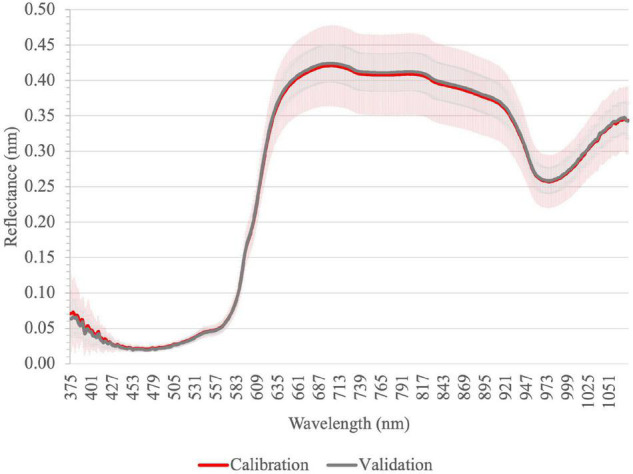
Average reflectance spectra of intact tomato fruit samples for calibration and validation in Vis-NIR by ASD; vertical bars represent the standard deviation (calibration *n* = 99; validation *n* = 33).

Figure 1 shows the visual representation of reflectance spectra of all intact tomato fruit samples obtained by ASD HH2 device. The profiles present broad but identifiable bands, ascribable to the contributions of the main constituents of the food matrix such as water and sugar. Reflectance value is below 0.1 from 400 to 575 nm as previously detected by ElMasry and Sun (52), including an intense absorption peak between 450 and 475 nm as found by Ciaccheri et al. (53). Above 560 nm, reflectance values rose sharply because of the red coloration of ripened fruits (54). The variability of spectra was the highest in the Vis-NIR region between 650 and 930 nm. The reflectance maximum was measured between 700 and 705 nm, as found by Clément et al. (52). In the NIR, there was a local absorption maximum at 976 nm.

**FIGURE 2:**
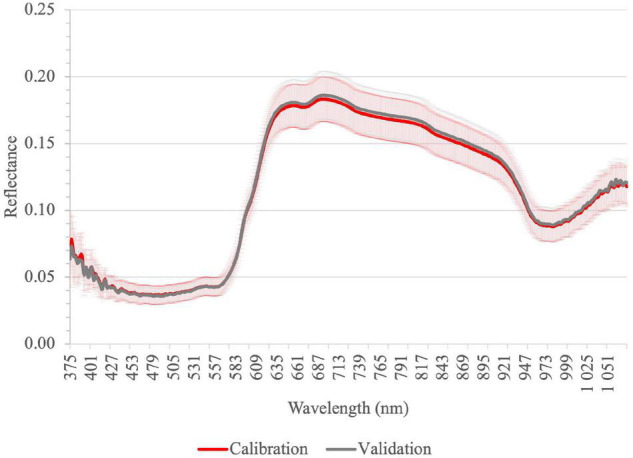
Average reflectance spectra of homogenized tomato fruit samples for calibration and validation in Vis-NIR by ASD; vertical bars represent the standard deviation (calibration *n* = 144; validation *n* = 48).

Figure 2 shows average reflectance spectra of all tomato homogenate samples for calibration and validation dataset in the range 375–1,075 nm. The results showed some absorbance peaks due to the vibration of O–H, C–H, and N–H bonds, which are related to inner fruit compositions such as sugars and acids. The absorption in the visible spectra is due to the fruit pigments such as chlorophyll, β-carotene, and lycopene. The highest bands in the VIS region (peaks at 550 and 607 nm) are because of the absorption of the chlorophyll, β-carotene, and lycopene similar to the results described previously (55). Yellow (570–590 nm), orange (590–620 nm), and red (620–750 nm) regions of reflectance spectra correlated well with tomato fruit color (56).

**FIGURE 3:**
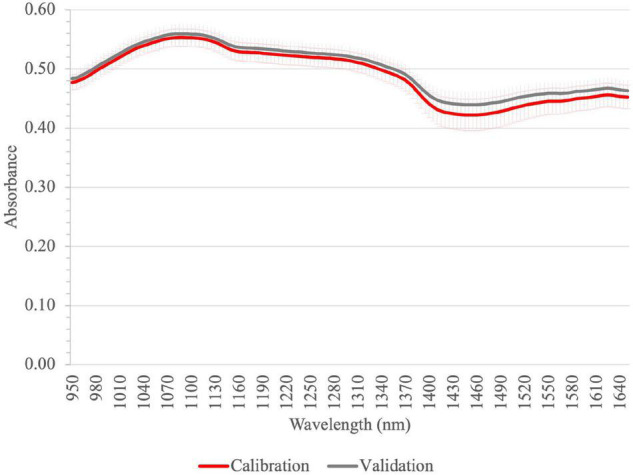
Average absorbance spectra of homogenized tomato fruit samples for calibration and validation in SWIR by Perten; vertical bars represent the standard deviation (calibration *n* = 144; validation *n* = 48).

The average absorbance spectra of the homogenized tomato fruit samples in the SWIR region measured by Perten can be seen in Figure 3. The highest bands (peaks at 1,095 nm) were in the SWIR region, which are due to the C–H, O–H, and N–H bonds (30, 57). The typical absorption bands related to the high water content of tomato samples can be seen around 950 and 1,450 nm, and a sugar-related peak appears also in the spectrum around 1,100 nm. Similar absorption bands were reported in the studies of tomato fruits and nectarines (58).

### Reference Values in Tomato Samples

To produce good quality paste of tomatoes, they need to be harvested at red ripe stage of fruits, with the highest possible DM content. Immediately after the spectra were measured, SSC measurements were performed using homogenized fruit samples. The samples were then frozen as the high-performance liquid chromatography (HPLC) capacity for lycopene determination did not allow simultaneous measurement of all samples. To make our models as general as possible, 25 varieties harvested over 2 years from three regions of Hungary were included in the samples.

**FIGURE 4:**
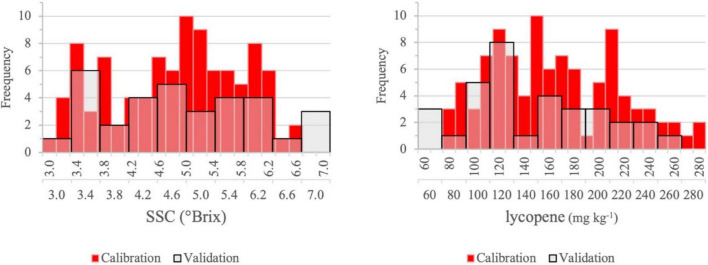
Distribution of SSC and lycopene content of intact tomato fruit for the calibration (*n* = 99) and validation (*n* = 33) sets.

**FIGURE 5:**
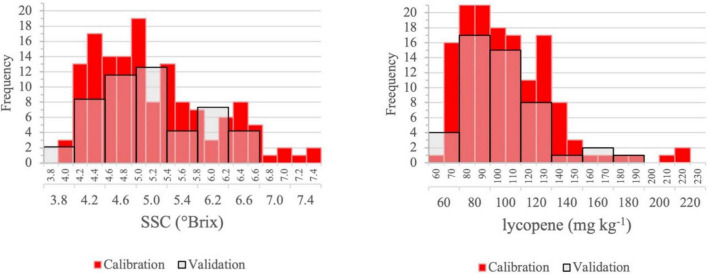
Distribution of SSC and lycopene content of homogenized samples for the calibration (*n* = 144) and validation (*n* = 48) sets.

Tomato fruit SSC is the first and lycopene content is the second most important quality attribute for the processing industry. For both properties, the distributions of the reference values in the calibration and validation set were comparable. The sequences of validation samples were designed to represent the characteristics of the calibration sequences. Samples were selected by genotype, by treatment combination, with three from the four replicates for calibration and one for validation (Figures 4, 5). In the figures, the transparent bars indicate the number of validation samples that contained fewer categories than the calibration samples. Values below 4°Brix have been found in the calibration samples, which would limit the opportunities of profitable processing and imply price reduction for the grower when measured at delivery. Processors expect a high SSC because the lower the water content of the raw material, the lower the cost of concentration.

Table 2 represents the average SSC and lycopene content of intact tomato fruits and homogenates used for calibration and validation. The parameters of the sample population selected for calibration and validation only slightly differed. Since the samples of intact and homogenized fruits were from two consecutive years, the higher SSC can be explained by the effect of seasonal variation according to our previous studies (59, 60). The effect of higher temperature on lycopene content is larger and opposite to that of SSC, which may have been caused by extreme high-temperature events in 2020 (59, 61).

**TABLE 2:** SSC and lycopene content of intact tomato fruits and homogenized samples in the calibration and validation sets.

	Total (*n* = 132)			Calibration (*n* = 99)			Validation (*n* = 33)		
Fruit	Range	Mean	*SD*	Range	Mean	*SD*	Range	Mean	*SD*
SSC (°Brix)	3.07–6.70	4.80	0.96	3.07–6.60	4.80	0.18	3.20–6.70	4.81	0.35
Lycopene (mg kg^–1^)	79.4–287.5	167.9	51.2	81.6–287.5	168.5	9.97	79.4–282.2	166.2	18.2

	**Total (*n* = 192)**			**Calibration (*n* = 144)**			**Validation (*n* = 48)**		
			
**Homogenate**	**Range**	**Mean**	***SD***	**Range**	**Mean**	***SD***	**Range**	**Mean**	***SD***

SSC (°Brix)	3.85–7.41	5.17	0.77	3.96–7.41	5.20	0.13	3.85–6.49	5.09	0.11
Lycopene (mg kg^–1^)	63–223	109.4	28.5	63.0–223.0	110.5	4.85	73.0–181.0	106.0	4.01

### Calibration and Validation

Both instruments were used to perform reflectance and absorbance measurements on homogenized samples, but the intact fruits could only be measured with the ASD instrument as they do not fit in the Perten sample tray due to their size.

### Soluble Solid Content Prediction

The results of SSC showed reliable correlation coefficient of cross-validation (*R*^2^ = 0.68) originated from raw reflectance of intact fruits and absorbance preprocessed by MSC (*R*^2^ = 0.72) with RMSECV 0.58 and 0.59, respectively in Vis-NIR spectra. In this spectral range, the absorbance data gave the smallest error (RMSECV 0.43) for the homogenized samples, which could not be improved by SNV (*R*^2^ = 0.58; RMSECV 0.43). The predictive capability of the SWIR spectrum for SSC gave the best results when using the first derivative of reflectance spectra, better than in the Vis-NIR (*R*^2^ = 0.61), and with lower error (RMSECV = 0.41).

**FIGURE 6:**
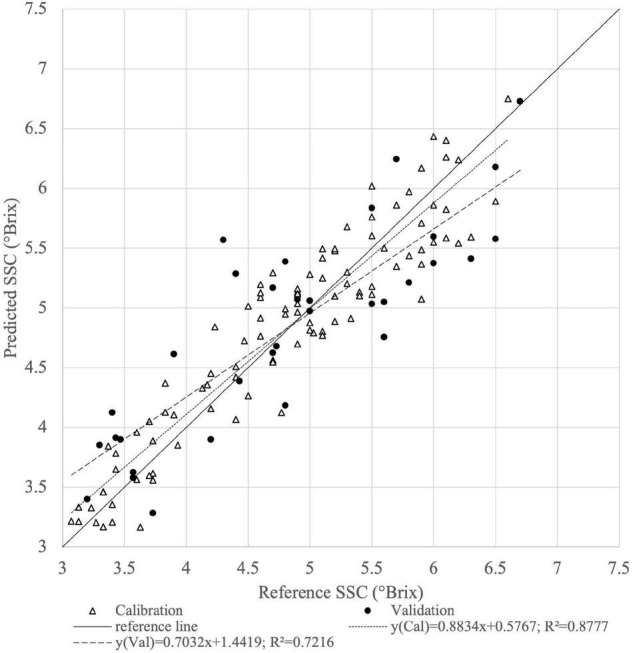
Calibration (Cal) and validation (Val) set of reference (*n* = 99) vs. predicted (*n* = 33) SSC of intact tomato fruits derived from the best PLSR model from absorbance of Vis-NIR spectra with MSC preprocessing.

Figure 6 shows the scatter plot of measured and predicted SSC using Vis-NIR PLSR models in the calibration and validation set of intact fruits by ASD. The best correlation was performed using the absorbance data + MSC spectral transformation of the samples for the determination of SSC agreed with others (32). The statistical parameters of prediction were *R*^2^ = 0.7216 and RMSECV = 0.59°Brix.

**FIGURE 7:**
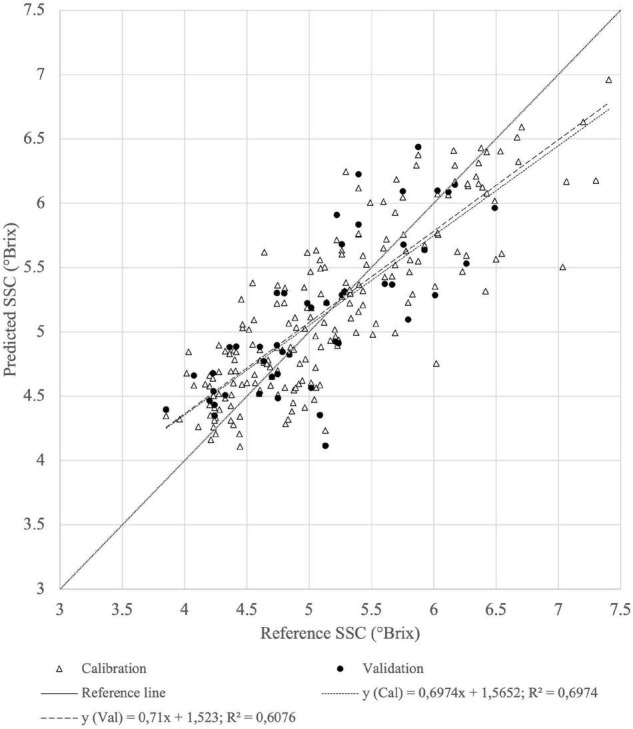
Calibration (Cal) and validation (Val) set of reference (*n* = 99) vs. predicted (*n* = 33) SSC of tomato homogenates derived from the best PLSR model from reflectance of SWIR spectra with first derivative preprocessing.

The correlation coefficient (*R*^2^ = 0.6821) and error (RMSECV = 0.58) of the first derivative of absorbance in homogenized fruit samples are only slightly different from the reflectance results (Figure 7), as has been described by others (51). Based on the graphical representations of the SSC calibrations and validations, the reflectance-based SWIR spectrum seems to be a better prediction method for homogenized samples.

Although DM is of major importance in tomato physiological research (62), the DM content of the fruit is closely related to its SSC (63). Therefore, SSC has been widely used in practice for the grading of raw tomatoes as it requires simpler sample preparation, cheaper devices, and less labor (4).

There are several difficulties to estimate SSC of tomato fruits non-destructively by spectral characteristic. Tomato fruits have a low SSC, which makes it more difficult to make a reliable prediction compared to fruits with a higher DM content (64). Especially, NIR-SSC predictions were heavily influenced by the correlation of inner and outer mesocarp SSC, which varied during fruit development (35).

Usually, the range of SSC in tomato fruit from the same cultivar and the same growing condition were relatively limited, so fruits using from different cultivars and production sites to achieve a wide range of fruit SSC are recommended for model development (65, 66). Therefore, we aimed to include more varieties and growing sites in the experiment to analyze a more representative sample population.

The scientific evidence generally agrees that Vis-NIR spectroscopy can be used to assess SSC in intact, thin-skinned fruit and is already being used in commercial practice. It is also expected that models based on transmittance will be more reliable in predicting SSC than models based on reflectance or absorbance as these methods are sensitive to surface reflectance variation (35, 67).

### Lycopene Content Prediction

**FIGURE 8:**
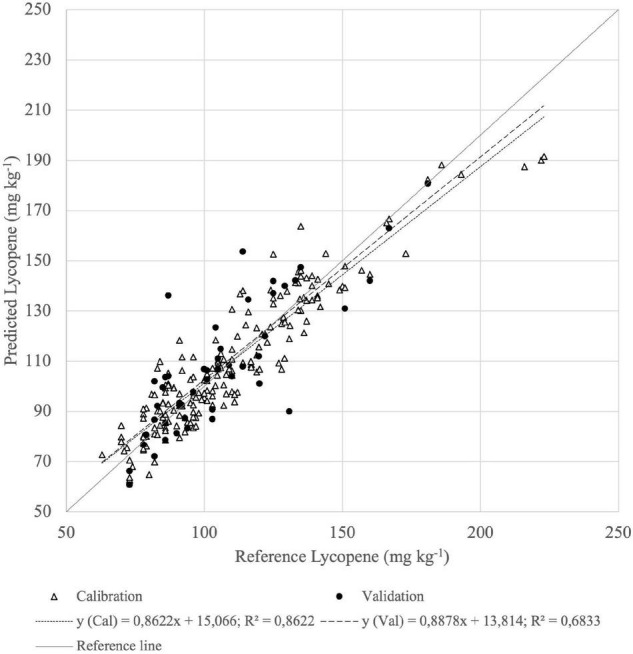
Calibration (Cal) and validation (Val) set of reference (*n* = 144) vs. predicted (*n* = 48) lycopene content of homogenized tomato fruit samples derived from the best PLSR model from absorbance of Vis-NIR spectra.

According to PLSR, based on Vis-NIR absorbance spectra, the best lycopene prediction of fruits resulted with MSC. The correlation coefficient of cross-validation was *R*^2^ = 0.46 and RMSECV = 39.6 mg kg^–1^ (Table 1). The best calibration of homogenate absorbance spectra by ASD had a more reliable predictive capability, RMSECV = 15.07 mg kg^–1^, where the correlation coefficient was *R*^2^ = 0.68 (Figure 8).

**FIGURE 9:**
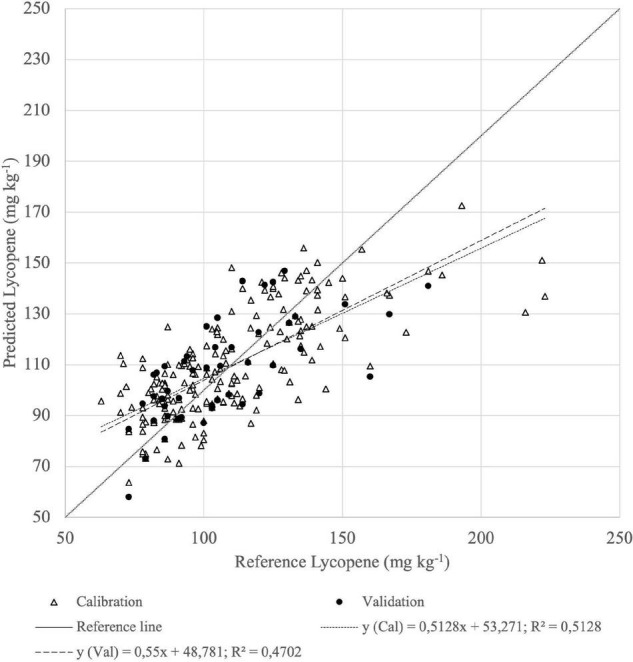
Calibration (Cal) and validation (Val) set of reference (*n* = 144) vs. predicted (*n* = 48) lycopene content of homogenized tomato fruit samples derived from the best PLSR model from absorbance of SWIR spectra with first derivative preprocessing.

The predictive ability in the SWIR range (*R*^2^ = 0.47; RMSECV = 17.95 mg kg^–1^) was lower for lycopene than in the Vis-NIR range, as represented in Figure 9.

The concentration of lycopene is not homogeneous in the fruit of tomatoes, being highest under the skin and much lower in the rest of the fruit (68), and recently bred tomato varieties with high lycopene content require more accurate methods to quantify lycopene content (69).

Lycopene content is well defined by non-destructively measured color values of tomato fruits, which in turn is highly dependent on variety and ripeness (56, 70, 71). VIS reflectance spectra can therefore be readily used to assess the main carotenoid in intact tomato fruit (72). Further investigation of NIR spectra on additional varieties to evaluate carotenoids in intact tomato fruit may help to develop more robust models (72, 73).

## Conclusion

Vis-NIR spectroscopy is a rapid tool to assist the industry or the laboratory for the estimation of the quality of raw or homogenized tomato fruits.

The use of multiplicative scattering correction and the first derivative were efficient preprocessing techniques for the validation and resulted in the most accurate estimation models of ingredients in tomato. Calibration models of raw absorbance from Vis-NIR spectra resulted in reliable prediction of intact fruits’ SSC (*R*^2^_*VAL*_ = 0.72), but SWIR spectral instrument produced lower RMSECV (0.41°Brix). Raw absorbance by Vis-NIR spectral range resulted slightly lower RMSECV of homogenate lycopene content (15.07 mg kg^–1^) comparing model of absorbance with first derivative in SWIR range (17.95 mg kg^–1^).

Combination of the two techniques (spectral range) could result in a more accurate calibration model for intact berries, which could be used to select raw material before the processing and for monitoring its homogenate in analytical laboratory or during the quality check of incoming raw material at the receiving area of processing plant. More homogeneous samples would result in a more accurate calibration, but this would not be conducive to the wide applicability of these models in practice. Vis-NIR spectroscopy appears to be a rapid and cost-effective technique compared to laboratory analytics, but using raw spectra requires a high level of skill because preprocessing is necessary. Traditional chemometric methods are time-consuming with higher cost and environmental impact, especially for lycopene analytics. The accuracy of the prediction obtained in this study indicates that Vis-NIR spectroscopy offers a useful method for quick and convenient evaluation of quality traits of tomato fruit. Further studies may give perspective to obtain better calibration models involving middle infrared range with more diverse sample population in accurate predictive capability.

## Data Availability Statement

The original contributions presented in this study are included in the article/supplementary material, further inquiries can be directed to the corresponding author.

## Author Contributions

ZP and MÉ: conceptualization and writing—original draft preparation. ST, HD, PS, and GaP: methodology and investigation. MÉ: software, data curation, and visualization. MÉ, ZP, and ST: validation. LH: resources and funding acquisition. ZP and GáP: writing—review and editing. GáP and LH: supervision. All authors have read and agreed to the published version of the manuscript.

## Conflict of Interest

GáP, GaP, and PS were employed by Univer Product PLC. The remaining authors declare that the research was conducted in the absence of any commercial or financial relationships that could be construed as a potential conflict of interest.

## Publisher’s Note

All claims expressed in this article are solely those of the authors and do not necessarily represent those of their affiliated organizations, or those of the publisher, the editors and the reviewers. Any product that may be evaluated in this article, or claim that may be made by its manufacturer, is not guaranteed or endorsed by the publisher.
